# Surgical Treatment Options for Giant Cell Tumors of Bone Around the Knee Joint: Extended Curettage or Segmental Resection?

**DOI:** 10.3389/fonc.2019.00946

**Published:** 2019-09-24

**Authors:** Hongbo He, Hao Zeng, Wei Luo, Yupeng Liu, Can Zhang, Qing Liu

**Affiliations:** ^1^Department of Orthopaedics, Xiangya Hospital, Central South University, Changsha, China; ^2^Department of Spine Surgery, The Second Xiangya Hospital, Central South University, Changsha, China

**Keywords:** giant cell tumors of bone, extended curettage, segmental resection, oncological prognosis, functional prognosis

## Abstract

**Aims:** This study aimed to compare and evaluate the oncological and functional prognosis of two surgical approaches for giant cell tumor of the bone (GCTB) around the knee joint and provide worthy suggestion for clinical treatment.

**Patients and Methods:** This study included 93 patients, who were divided into the extended curettage (EC) group and segmental resection (SR) group. Relevant preoperative and postoperative data were collected, oncological and functional prognosis were evaluated, and postoperative complications of the two groups were analyzed. Local recurrence was assessed via clinical and radiological tests. Functional prognosis was evaluated using the Musculoskeletal tumor Society (MSTS) scoring system.

**Results:** The EC group had 69 patients; it included 57 primary cases and 12 recurrent cases. The SR group had 24 patients (12 men and 12 women; mean age, 34.9 years), including 15 primary cases and 9 recurrent cases. In this study, six cases (6.5%; EC group, 5 cases; SR group 1 case) recurred within 18 months postoperatively. There was a significant difference in the mean MSTS score between the two groups (*p* < 0.001). Nononcologic complications occurred frequently in the EC group than in the SR group (28.0 vs. 16.7%), but no complications had serious consequences, and the functional prognosis was not affected.

**Conclusion:** EC and SR for GCTB around the knee joint can achieve satisfactory oncological prognosis, but we should individually select the most suitable surgical method according to Campanacci grade, age, and long-term complications of patients and consider the functional prognosis to ensure excellent oncological prognosis.

## Introduction

A giant cell tumor of bone (GCTB) is a primary bone tumor with potential invasion, local recurrence, and low probability of distant metastasis (1). Studies have shown that GCTB accounts for 5–7% of all primary bone tumors and 20% of all benign bone tumors (2). Its incidence in China was about 14–20%, which was higher than 5–8% in other eastern countries (3). GCTB tends to occur in people aged 20–40 years, accounting for 60–75% of all patients (3), and GCTB occurs in the meta-epiphyseal area of the limbs and in the around knee joint at around 50–65% of the whole body, especially in the distal femur and proximal tibia.

GCTBs grow in an expansive manner and easily penetrate the cortex of the bone or even cause pathological fracture. Although they rarely expand into the articular cavity, they invade the subchondral bone, which seriously affects knee joint function (4). These factors lead to an embarrassing situation during treatment because the knee joint is the main load-bearing joint of the lower limbs and has high functional requirements. The therapeutic purpose of GCTB around the knee joint is to reduce its recurrence rate and maximize the recovery of joint function, while reconstructing the integrity of bone structure and articular surface, as well as obtaining normal biomechanics and preventing the occurrence of long-term osteoarthritis (5–7).

There is still controversy about the surgical treatment of GCTB in the around knee joint. How to achieve a balance between completely removal of tumors to reduce recurrence and preservation of knee joint function as much as possible was the linchpin for clinicians to balance. The surgical treatment of GCTB in the around knee joint mainly includes curettage and bone grafting (3, 8), extended curettage (EC) and cement filling (1, 9), segmental resection (SR) and artificial prosthesis reconstruction (6, 10). Although these methods have achieved certain results in the treatment of GCTB, some problems occur, such as local recurrence (11, 12), secondary osteoarthritis (1, 13), cartilage surface collapse (14), artificial prosthesis loosening and infection (6, 10), which require deep focus and improvement.

Therefore, this study aimed to analyse the correlation between the choice of surgical treatment for GCTB around the knee joint and the prognosis of oncology and limb function through a single-center retrospective cohort study to provide a valuable reference for surgical treatment of GCTB around the knee joint.

## Patients and Methods

### Patients

**Figure 1:**
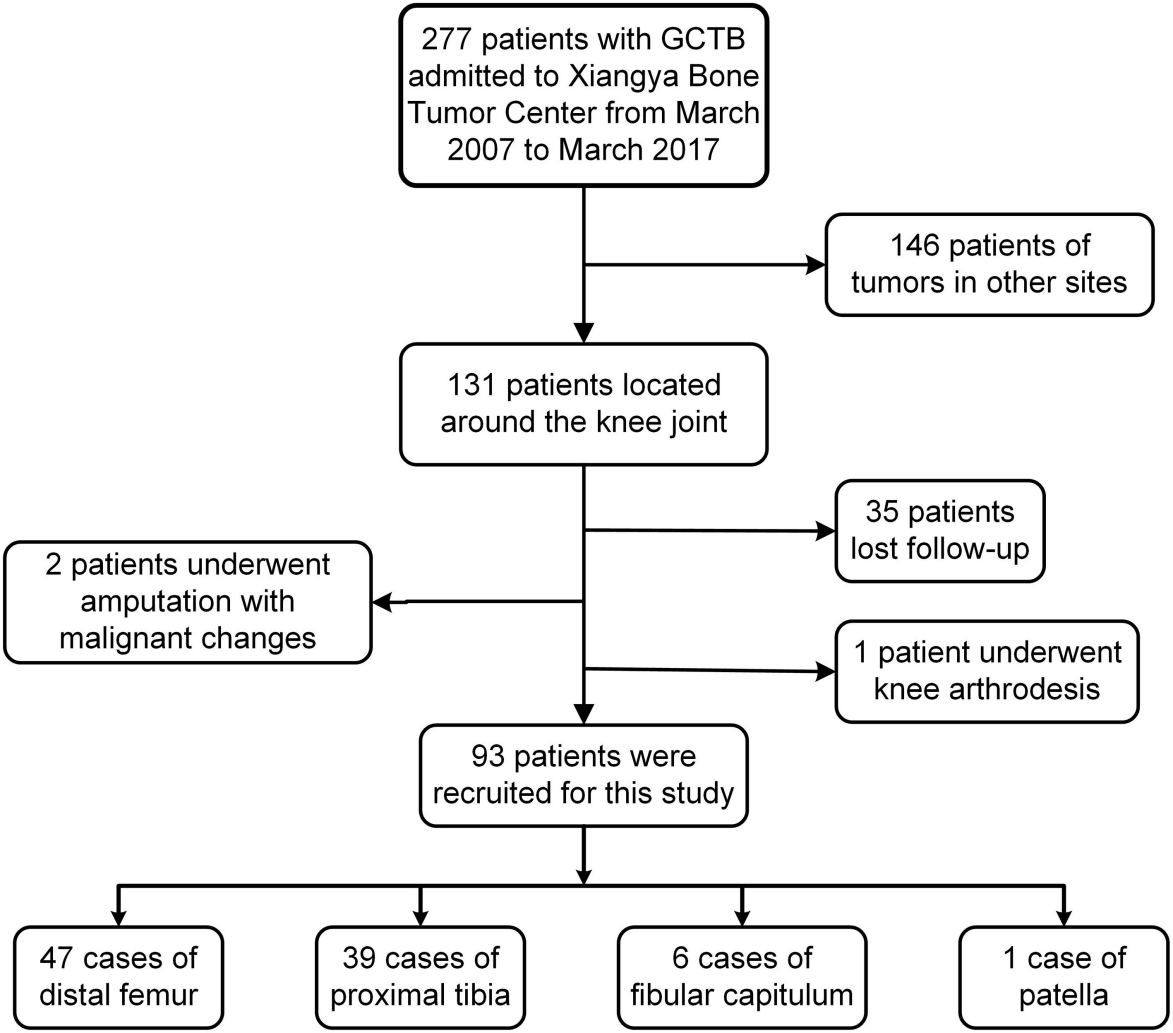
Flow charts of patients included in this study.

Data of 277 GCTB patients who were treated at a single center from March 2007 to March 2017 were retrospectively collected. The inclusion criteria were as follows: GCTB located in the around knee joint, histopathological diagnosis of benign GCTB, surgical treatment with limb salvage, and postoperative follow-up of more than 24 months with integrated data. According to the above criteria, a total of 131 patients with GCTB located around the knee joint were retrieved. Among them, 35 patients lost follow-up, two had amputation due to malignant changes, and one received knee arthrodesis. Finally, 93 patients were enrolled in the present study (Figure 1). Radiography, computed tomography (CT), and magnetic resonance imaging (MRI) were performed preoperatively to determine the extent of tumor invasion and pulmonary metastasis. Lesions were graded according to Campanacci classification of imaging (15). Meanwhile, preoperative puncture biopsy was performed to confirm the diagnosis of GCTB. Patients were divided into the EC group and SR group.

This study was approved by Xiangya Hospital Ethics Committee, and written informed consents were obtained from the patients or their legal guardians.

### Procedure

**Figure 2:**
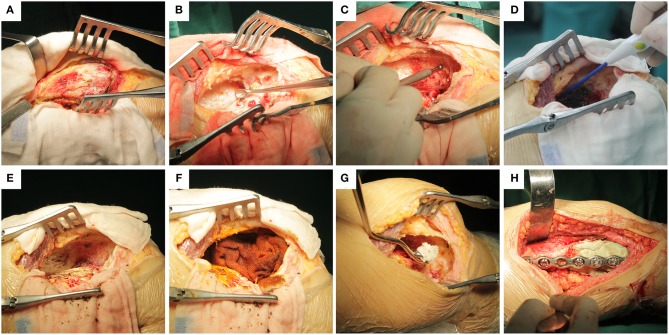
Specific implementation steps of extended curettage: **(A)** Fenestration from the eccentric cortex of the lesion and protecting peripheral tissues with wet saline gauze. **(B)** Scraping the lesion thoroughly with different types of curette. **(C)** Grinding residual bone ridges in cavity with high-speed burr. **(D)** Cauterization of the cavity wall with electrotome. **(E)** Scraping blackened bones and rinsing thoroughly again. **(F)** Cotton balls with iodine tincture were used to smear the cavity wall for 3 min before rinsing again. **(G)** Transplantation of allogenic cancellous bone into the subchondral bone at least 1 cm thick. **(H)** The remaining cavity was filled with cement, and finally, internal fixation was performed.

EC was performed as follows. The fenestration from the eccentric cortex of the lesion was sufficiently large to avoid opening the joint capsule (Figure 2A). To protect peripheral tissues, wet saline gauze was used before fenestration to reduce tumor cell implantation. Different types of curettes were used to scrape the tumors thoroughly, and the cavity was rinsed with sterilize water (Figure 2B). Then, the residual bone ridges in the cavity were grounded with high-speed burr (Figure 2C); the range of grinding was 10 mm for the normal cancellous bone and 1 mm for the cortical bone (only residual bone ridges adjacent to the articular surface were removed). Afterwards, an electrotome was used to cauterize the cavity wall (Figure 2D), and the blackened bone was scraped again (Figure 2E). Iodine tincture (10% concentration) was applied meticulously using a surgical cotton ball and left for 3 min (Figure 2F). After which, the cavity was irrigated with sterile water again. After thorough curettage, the cavity was filled with cement and allograft. Allografts about 1 cm thick were transplanted to the side around the articular surface, and the patellar cavity was filled with allograft only (Figure 2G). The remaining cavity was filled with cement, and internal fixation was performed finally (Figure 2H).

SR was performed as follows: The surgical resection margin was determined by preoperative T1-weighted-enhanced images. The tumors were completely resected from normal peripheral tissues, while the common peroneal nerve and important vessels of the lower limbs (femoral and posterior tibial arteries) were protected during the surgery. After resection, bone defects were repaired with artificial prosthesis followed by soft tissue repair, but the fibula was an exception, where only the lateral ligament of knee joint was repaired, and the bone defect was not repaired.

Patients of the EC group were exempted from weight-bearing for 2 weeks, and they gradually began to perform non-weight, semi-weight, and full-weight bearing functional exercises alternately using crutches. Limbs of the patients in the SR group were fixed with plaster or braces for 4–6 weeks, and they gradually began to perform functional exercises, from half-load to full load with crutches.

### Follow-Up and Evaluation

Patients were followed radiographically every 3 months for the first 2 years after the surgery, every 6 months until the 5th year, and annually until the 10th year. The radiographs of the involved area and CT images of the chest were obtained to evaluate cancer prognosis. The prognosis of limb function was evaluated based on the last follow-up record and those of recurrent patients were based on the best functional records before recurrence. Evaluation tools used were as follows: the Musculoskeletal Tumor Society (MSTS) (16) Score was used to assess function. The visual analog scale (VAS) (17) was used for pain evaluation. Osteoarthritic change was evaluated by the Kellgren-Lawrence (K-L) grading system (18). Rejection, prosthesis loosening, periprosthetic fracture, and infection were also recorded.

### Statistical Analysis

Data were analyzed using SPSS software version 20.0 (IBM Corp., Armonk, NY), and measurement data were expressed as mean ± standard deviation. Multivariate and univariate Cox regressions were used to analyse risk factors of local tumor recurrence. Continuous variables were compared by one-way analysis of variance, and categorical variables were compared by chi-square test. *P* ≤ 0.05 was considered statistically significant.

## Results

### Patients

According to the statistical results of the data (Table 1), the EC group included 69 patients (37 men and 32 women), with the mean age of 36.3 (range, 17–65) years. The number of involved femur, tibia, and patella were 36, 32, and 1, respectively, and the average length of the lesion was 5.6 cm (range, 2.6–8.8 cm). The preoperative Campanacci grades were I, II, and III in 7, 33, and 29 cases, respectively. There were 57 primary cases and 12 recurrent cases (recurrence after initial treatment in other centers). There were 18 preoperative pathological fractures.

**Table 1:** Patient demographics.

**General information**	**EC group**	**SR group**
Mean age (sd)	36.3 (12.5)	34.9 (9.9)
Gender, *n* (%)		
M	37 (53.6%)	12 (50.0%)
F	32 (46.4%)	12 (50.0%)
Lesion length (mm, mean ± SD)	5.6 ± 1.2	7.2 ± 1.3
Lesion location, *n* (%)		
Femur	36 (52.2%)	11 (45.8%)
Tibia	32 (46.4%)	7 (29.2%)
Fibula	0	6 (25.0%)
Patella	1 (1.4%)	0
Campanacci grade, *n* (%)		
I	7 (10.1%)	0
II	33 (47.8%)	0
III	29 (42.0%)	24 (100%)
Prior surgery, *n* (%)	12 (17.4%)	9 (37.5%)
Pathological fracture, *n* (%)	18 (26.1%)	14 (58.3%)

The SR group included 24 patients (12 men and 12 women), with the mean age of 34.9 (range, 17–52) years. In this group, 11 femurs, 7 tibias, and 6 fibulas were examined. The average length of the lesion was 7.2 cm (4.3–10.2 cm). All cases were of Campanacci grade III. There were 15 primary cases and 9 recurrent cases. There were 14 cases of preoperative pathological fracture and two cases of pulmonary metastasis.

### Oncology Prognosis

**Figure 3:**
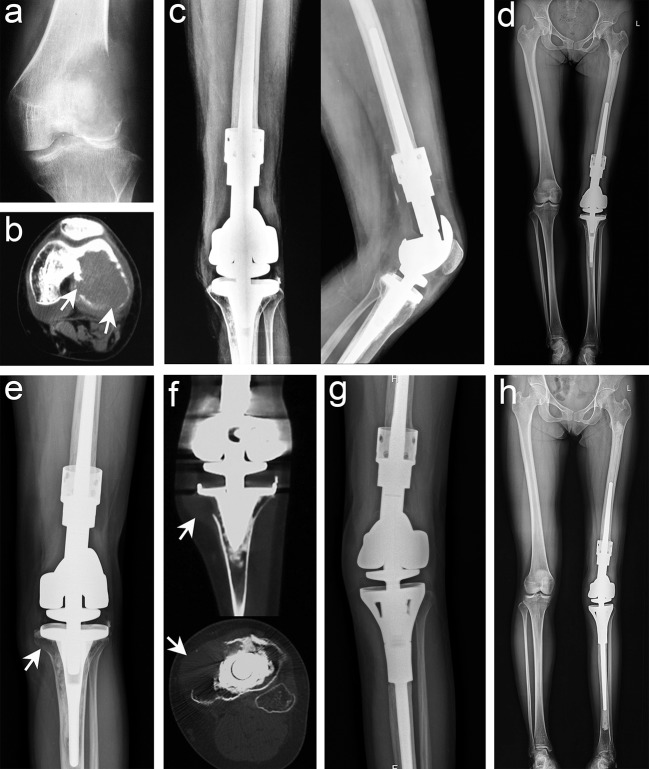
Typical imaging manifestations of patients with recurrence treated with segmental resection. **(a)** Anteroposterior radiographs of recurrence after curettage in other hospitals. **(b)** Computed tomography (CT) image showing that the lesion had penetrated the bone cortex and involved the intercondylar fossa and the posterior part of the joint. **(c,d)** Anteroposterior, lateral, and total length of lower limb radiographs after segmental resection and artificial prosthesis reconstruction. **(e)** Osteolytic lesions were found in the proximal tibia 15 months after the operation. **(f)** CT scan confirmed that the lesion invaded the peripheral tissues. **(g,h)** Anteroposterior and total length on the lower limb radiographs after segmental resection was performed again and artificial prosthesis reconstruction (white arrow points to the lesion).

In this study, six cases (6.5%) of recurrence occurred within 18 months after surgery. There were five recurrence cases in the EC group, including three cases in the femur and two cases in the tibia, of which one was far from the articular surface and the four were around to the articular surface. Preoperative pathological fracture occurred in two cases, and two cases were transferred from another hospital as local recurrence. All five patients, including three cases with EC and two cases with SR, were reoperated. No recurrence or metastasis was found at the latest follow-up (Table 2). There was only one recurrence in the SR group, of which the patient had distal femoral recurrence *in situ* 11 months after curettage at other centers. We performed SR and artificial prosthesis reconstruction for the first time. Unfortunately, the patient had a recurrence at the tibia 15 months after the first operation in our hospital. We re-performed SR and artificial prosthesis reconstruction for patients after puncture biopsy with confirmed GCTB, and the latest follow-up was satisfactory (Figure 3). One of the two patients with preoperative pulmonary metastases underwent laparoscopic resection, and the other refused to undergo surgery and continued follow-up. No patients had secondary pulmonary metastases after operation. We found that the recurrence rate of the EC group was higher than that of the SR group, but the difference was not statistically significant (*p* = 0.597). Among the disease-related and demographic factors analyzed for their effects on recurrence, age, sex, lesion location, lesion length, pathological fracture, recurrence or not, Campanacci grade, etc., appear to have no significant effects on local recurrence (Table 2).

**Table 2:** Prognostic comparative statistics.

**Variable**	**EC group**	**SR group**	***P*-value**
Duration of follow-up (month)	67.8 ± 38.7	70.9 ± 27.7	0.720
Mean pre-op VAS (sd)	4.5 (1.9)	5.5 (1.8)	0.033
Mean post-op VAS (sd)	0.3 (0.5)	1.0 (0.8)	0.000
Mean MSTS score (sd)	28.2 (1.8)	26.5 (1.4)	0.000
Local recurrence, *n* (%)	5 (7.2%)	1 (4.2%)	0.597
Complication, *n* (%)			
Osteoarthritis	6 (8.6%)	0	
Rejection reaction	17 (24.6%)	0	
Joint stiffness	5 (7.2%)	4 (16.7%)	0.006
Fracture	1 (1.4%)	0	
Reoperation, *n* (%)	5 (7.2%)	1 (4.2%)	0.597

### Limb Function Prognosis

A significant difference was found in the mean MSTS score between the two groups (EC group, 28.2 points; range, 24–30 points, 95% CI 27.8–28.5; SR group, 26.5 points; range, 27.7–28.5 points, 95% CI 0.58–0.94; *p* < 0.001). All patients in this study resumed normal activity after operation. Of the 93 patients, 82 patients (88.2%) returned to their full level of preoperative function and had excellent functional recovery at the latest follow-up. Pain symptoms improved significantly. Moreover, 84 patients (90.3%) returned to their previous jobs (Table 2).

### Complication

Nononcologic complications occurred frequently in the EC group than in the SR group (28.0% [29/69] vs. 16.7% [4/24]). In the EC group, six patients had secondary osteoarthritis (five cases with K-L grade 2 and one case with K-L grade 3). Symptoms of osteoarthritis occurred at a mean of 33 months after surgery, but fortunately, these patients did not need surgical treatment for the time being. Seventeen patients developed mild rejection within 1 week after operation, and symptoms disappeared after oral administration of low-dose hormones. Five patients developed joint stiffness, and the patient with patellar lesion developed fracture after complete healing of the lesion. In the SR group, joint stiffness developed in four patients, while other complications were not observed. Postoperative fracture, infection, and failure of internal fixation were not observed in both groups.

## Discussion

GCTBs in the around knee joint were a clinical challenge in orthopedics, as the knee joint is the most important weight-bearing joint with high functional requirements. Furthermore, biologically, GCTB showed expansive growth, which can easily break through the bone cortex and even cause pathological fracture (1, 3, 4, 7). Although it rarely spread into the articular cavity, subchondral bone involvement was not uncommon, which can have a serious effect on the function of the knee joint. The treatment of GCTB in the around knee joint should follow the principle of thoroughness and functionality. Thus, it is necessary to thoroughly remove the tumor tissue to recover joint function. Therefore, how to achieve a balance between radical removal of tumors to reduce recurrence and preserve knee joint function is very important for GCTB around the knee joint.

Considering that the main surgical methods were EC (4, 19) and SR (5–7), it is of great practical significance to analyse and evaluate the efficacy of EC and SR of GCTB around the knee joint. The recurrence rate of GCTB in curettage and bone grafting was 40–60% because of insufficient surgical margin (3). Consequently, in GCTB treatment, some authors used physical or chemical methods such as high-speed burr (20), ethanol (21), phenol (22), liquid nitrogen (23), and other physical or chemical methods to expand the surgical curettage boundary, followed by cement repair of bone defects to achieved satisfactory results.

**Figure 4:**
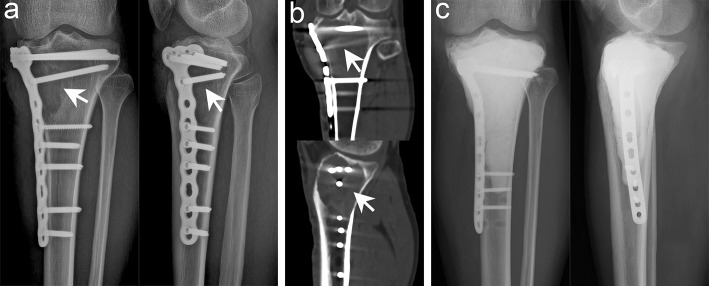
Typical imaging manifestations of patients with local recurrence treated with extended curettage. **(a)** Anteroposterior and lateral radiographs showed that the proximal and lateral parts of the right tibia had obvious osteolytic foci at the original site. **(b)** Computed tomography (CT) scan confirmed low-density osteolytic changes in the original site. **(c)** Extended curettage, cement filling, and subchondral bone grafting were performed after relapse of GCTB was confirmed by biopsy (white arrow points to the lesion).

In the present study, we used high-speed burr, electrotome cauterization, and iodine tincture to treat the tumors successively, and we achieved satisfactory oncological prognosis (Figure 4). Only five cases (7.2%) had recurrence, which was significantly better than that reported in previous studies (3, 24). High-speed burr can grind the bone ridge in the cavity; hence, it was convenient to remove the residual tumor tissue around the bone ridge. Electrotome cauterization can sweep the tumor wall extensively, and finally, iodine tincture can denature the protein and cause coagulation necrosis of the cells. Consequently, successive combined treatment can treat the lesions from an omni-direction and multi-angle to reduce recurrence.

Although EC can achieve excellent oncological prognosis, we had summed up some experience from recurrent cases: The window must be large enough for curettage under direct vision, and application of sterile oral endoscopy may help in the removal of small lesions in blind visual field. The use of adjuvant therapy should focus on the treatment of articular lateral tumors to preserve the subchondral bone as much as possible and to achieve the goal of EC. For patients with pathological fracture and recurrence, as long as the fracture line or lesion did not involve the articular cartilage, it can still be treated by EC, and the patient's oncological prognosis was still satisfactory.

The repair of bone defect after EC was also a focus of current clinical controversy. Previous studies have confirmed that cement has many benefits in repairing bone defects after EC of GCTB: The heat released during cement solidification can kill the residual tumor cells in the cavity to achieve the effect of extended curettage (25). It provides strong support to allow early weight-bearing. Cement filling was suitable for all shapes and sizes of bone defects. Recurrence can be detected early on X-ray imaging (7). Although cement provided many satisfactory benefits, due to the difference in elastic modulus between the cement and normal bone, the surrounding bone can be gradually absorbed after stress and lead to the loosening of the cement, resulting in the “ball effect,” so it is often necessary to use internal fixation when filling with cement. Considering that cement directly adheres to the subchondral bone or articular cartilage, its local thermal effect and stress concentration on the subchondral bone and articular surface can easily lead to cartilage damage, which increases the risk of intra-articular fracture and early osteoarthritis (26). Therefore, subchondral bone grafting was often used as a buffer zone to avoid the harmful effects of bone cement (4, 27, 28). Radev et al. (28) performed finite element analysis and found that as long as there was at least 3-mm uniform cancellous bone above the cement, the thermal effect of the cement will not endanger the articular cartilage and subchondral bone. We used cement filling and subchondral bone grafting (5–10 mm) to repair bone defects in the EC group (Figure 3), and the cement was fixed. Only five patients were found to have secondary early osteoarthritis during follow-up, and surgical treatment was not required. Therefore, we have also proven that subchondral bone grafting can avoid the direct damage of cement to cartilage and reduce the incidence of postoperative complications without affecting the recurrence rate, which was consistent with that reported in previous studies (7, 29, 30).

**Figure 5:**
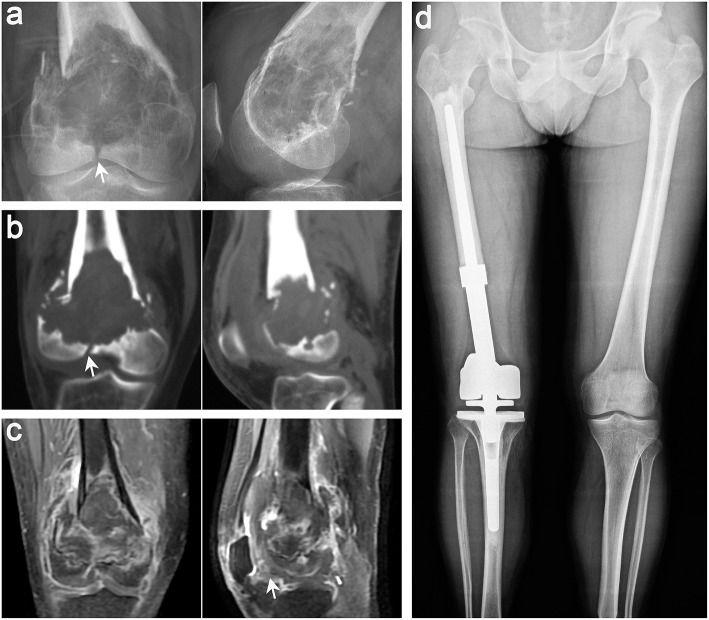
Typical imaging features of a 38-year-old man with GCT in the distal femur on the right side. Anteroposterior and lateral radiographs **(a)**, CT scan **(b)**, and MRI **(c)** of the knee joint showed that the patient had pathological fracture that involved the articular surface, which was defined as Campanacci III GCT. **(d)** Anteroposterior radiographs showing that both lower limbs were equal in length and the prosthesis was stable 15 months after segmental excision and artificial prosthesis reconstruction (white arrows indicate key points).

SR, as an excellent surgical method for oncological prognosis, was recommended for GCTB of the proximal fibula (31), distal radius (32), and part of Campanacci grade III (Figure 5) (5, 29, 30). Medellin et al. (5) reported a lower recurrence rate in patients with Campanacci grade III using SR than using EC through a comparative study, and the results were further confirmed by Renard et al. (33). Deheshi et al. (34) retrospective analysis of limb salvage treatment for GCTB in weightbearing long bones revealed that SR were the preferred treatment for patients with severe joint destruction or dislocation, comminuted or intra-articular fractures. It is also interesting that Balke et al. (35) have found that SR is more recommended for recurrent GCTB because it can achieve satisfactory oncological prognosis. So at this point, we can reach a consensus that SR can achieve better oncological prognosis in the surgical treatment of high-grade complex GCTB. But the pros and cons often coexist, although the oncological prognosis of patients undergoing SR was satisfactory, the functional prognosis of prosthesis replacement can not to ours heart's content, which was a mechanical reconstruction method had some deficiencies.

Prosthetic replacement can make patients recover joint function early without affecting appearance and provide good joint stability and range of motion, but long-term complications may engender a heavier burden on patients (5–7, 10). Through long-term follow-up study, Bus et al. (36) found that there were high mechanical and structural complications in artificial prostheses of knee joint, and the failure rate of implants would gradually increase with time. The cumulative rates of implant failure in 5, 10, and 15 years due to mechanical failure were 16.9, 20.7, and 37.9%, respectively. Franklin's (37) long-term study of the effects of cement prosthesis on periarticular tumors of the distal femur also found a high risk of revision, reoperation, and infection. These studies have confirmed that prosthesis reconstruction may result in mechanical and structural complications such as deep infection, aseptic loosening, fracture of prosthesis stalk, which seriously affect the life of prosthesis and the prognosis of patients' function. Considering the age of onset, we can predict that many patients will receive revision surgery due to various complications in the long term, which will increase the financial burden of patients and sacrifice more joint function.

**Figure 6:**
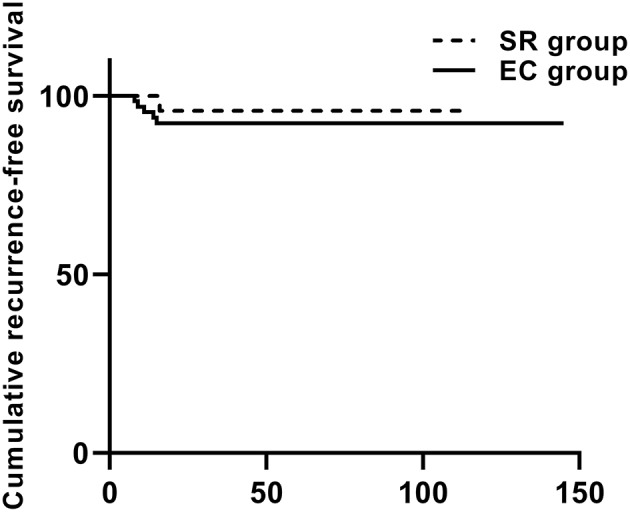
The cumulative recurrence-free survival in the Kaplan–Meier curve was based on local recurrence and 93 cases according to the type of surgery.

Through this study, we found that the treatment of GCTB around the knee joint seems to be appropriately conservative, giving more patients a joint salvage opportunity. Considering that GCTB mostly occurred in individuals aged 20–40 years, long-term function cannot be guaranteed by SR and prosthesis replacement. For patients with Campanacci grade III, we also recommended EC (unless a large mass of peripheral soft tissue was involved or a pathological fracture involves the articular surface). Even if the patient unfortunately relapses, we can use SR to make up for it. Both SR and EC can effectively reduce the recurrence rate (Figure 6), but we should balance the recurrence rate and postoperative complications comprehensively when selecting surgical methods for individual patients to maximize the functional prognosis of patients on the premise of guaranteeing excellent oncology prognosis.

We have also acquired some unique insights into the treatment of GCTB through this study: Oncological and functional prognosis should be regarded both as equally important in the treatment of GCTB around the knee joint. Complete removal of the lesion was the fundamental guarantee for oncology prognosis, and subchondral bone grafting was a good choice to avoid secondary early osteoarthritis. SR was recommended for patients with pathological fracture involving the articular surface and lesion that extensively invades the surrounding tissue. EC was still preferred for recurrence as long as the articular surface and peripheral tissue are not involved.

Our study should be interpreted in light of its limitations. Similar to many orthopedic oncology studies, our study was retrospective, the number of patients was limited, and the follow-up time of patients was inadequate for assessment of long-term complications. Additionally, the number of patients in the two groups varies greatly, so there are some biases in the statistical results, which may weaken the real validity of the results. Overall, this study comprehensively analyzed the efficacy of the two methods in the treatment of GCTB around the knee joint, confirmed the excellent oncological prognosis of EC and SR, and compared the functional prognosis of the two methods, which could provide correct guidance for the surgical treatment of GCTB around the knee joint.

In conclusion, EC and SR for GCTB around the knee joint can achieve satisfactory oncological prognosis, but we should individually select the most suitable surgical method according to Campanacci grade, age, and long-term complications of patients and take into account the functional prognosis to ensure excellent oncological prognosis.

## Data Availability Statement

The datasets generated for this study are available on request to the corresponding author.

## Ethics Statement

The studies involving human participants were reviewed and approved by the Research Ethics Committee of Xiangya Hospital. The patients/participants provided their written informed consent to participate in this study.

## Author Contributions

HH: conceptualization and design of the study, performed the surgical procedures, collected and analyzed the data, prepared the manuscript, and approved final version of the manuscript. HZ: analyzed the actigraphy data and approved final version of the manuscript. WL: performed the surgical procedures, critical revision the manuscript, and approved final version of the manuscript. YL: performed the surgical procedures, screened and included eligible patients, and approved final version of the manuscript. CZ: performed the surgical procedures, analyzed the data, and approved final version of the manuscript. QL: conceptualization and design of the study, data collection, statistical analysis, manuscript drafting and revision, and approved final version of the manuscript.

### Conflict of Interest

The authors declare that the research was conducted in the absence of any commercial or financial relationships that could be construed as a potential conflict of interest.

## Abbreviations

GCTB: giant cell tumor of bone
CT: computed tomography
MRI: magnetic resonance imaging
EC: extended curettage
SR: segmental resection
MSTS: Musculoskeletal tumor Society
VAS: Visual Analog Scale
K-L: Kellgren-Lawrence.
